# High adsorption capacity of phenol and methylene blue using activated carbon derived from lignocellulosic agriculture wastes

**DOI:** 10.1038/s41598-022-09475-4

**Published:** 2022-03-31

**Authors:** Haitham M. El-Bery, Moushira Saleh, Reem A. El-Gendy, Mahmoud R. Saleh, Safinaz M. Thabet

**Affiliations:** Advanced Multifunctional Materials Laboratory, Chemistry Department, Faculty of Science, Assiut University, Assiut, 71515 Egypt

**Keywords:** Engineering, Materials science, Environmental chemistry

## Abstract

The resources of clean water worldwide are very limited, and climate change is already affecting the available supplies. Therefore, developing a low-cost, highly efficient, and recyclable adsorbent to upgrade water quality has become an essential task. Herein, we report the fabrication of activated carbon (AC) adsorbents derived from lignocellulosic wastes. Both physical and chemical activation were investigated to modify the surface texture properties. The results indicated that increasing the activation temperature, whether physically or chemically, increases the specific surface area (S_BET_). On the contrary, increasing the amount of the chemical activating agent significantly decreases the S_BET_ values. The S_BET_ of 1771, 2120, and 2490 m^2^ g^−1^ were obtained for water vapor, K_2_CO_3_ and KOH, at activation temperatures of 950 °C, 800 °C, and 800 °C, respectively. Methylene blue (MB) and phenol were used as adsorbates for the adsorption experiment. Adsorption of methylene blue dye revealed the ability of the water activated carbon to remove more than 95% of the dye (100 ppm) within 5 min with an adsorption capacity of 148.8 mg g^−1^. For phenol adsorption, Several parameters were investigated, including initial concentration (50–250 ppm), pH (2–10), contact time (5–60 min), and temperature (25–45 °C). The highest adsorption capacity of phenol achieved was 158.9 mg g^−1^. The kinetics of adsorption of phenol was better described by pseudo-second-order reaction while the isotherm process using Langmuir model. This study presents a roadmap for conversion of lignocellulosic biomass waste into highly efficient porous carbon adsorbents.

## Introduction

Industrial wastewater contains high percentages of recalcitrant and carcinogenic pollutants such as phenolic compounds discharged from petroleum and petrochemicals industries^1–3^. Phenol concentration discharged in various industrial wastewaters could reach more than 1 ppm, due to its solubility in water^4^. The presence of phenolic compounds, as carcinogens, in fresh water streams has negative environmental and health effects. Because of its chemical and physical properties, it can disrupt the endocrine systems and poison aquatic life^5^. Consequently, the World Health Organization (WHO) established that the maximum permissible concentration of phenol in potable water does not exceed 1.0 ppb^6^. Therefore, the treatment of wastewater containing phenol is an intriguing research topic, which has preoccupied many researchers to ensure environmental safety during waste disposal^7^. Several ecofriendly methods can be used to get rid of phenol in aqueous solutions, for example adsorption^8^, chemical oxidation^9^, coagulation^10^, membrane filtration^11^ and others^12^. However, the adsorption method has advantages over the abovementioned methods, such as simplicity in operation and implementation, high efficiency, cost-effectiveness, and recyclability^13^.

The adsorption process has proven its practical ability to remove harmful pollutants using porous materials as adsorbents like activated carbon, metal–organic frameworks, and metal oxides^14–16^. Among these materials, activated carbon (AC); that describes carbonaceous materials with basic properties that are promising due to their porous structure, large specific surface area, and functionalized surface^17^. Many precursors were used to develop AC, which possesses high availability, cost-effective and harmless to the environment^18^. Among the different precursors used, lignocellulosic biomass is a benchmark in fabricating highly porous AC as it can be disposed of without environmental pollution^19^. Carbon materials resulting from the pyrolysis process at high temperatures have a porous nature^17^. However, in order to improve the surface area and surface functionalization, the activation process, either physically or chemically, is required^20^. The process of physical activation is often carried out in two steps. The precursor is burned at high temperatures in a neutral atmosphere; then the carbonized product is heated in an atmosphere of oxidizing gases such as water vapor and carbon dioxide at a temperature range of 800–1000 °C. On the other hand, the chemical activation process can be done by combining the precursors with the activated chemical agents before pyrolysis/activation. Based on what has been mentioned about the importance of the activation process on the pore structure and texture of the AC, which is closely associated with the pollutant removal. Several activation methods including, physical or chemical, have been conducted to convert lignocellulosic biomass waste into AC^21–27^. It was found that the activation processes could greatly enhance the physicochemical properties of the carbon materials e.g. surface area, and carbon yield. However, the effect of different activation processes on the properties of the prepared activated carbon has not been comprehensively studied. Accordingly, the main objective of the presented study is to apply an effective, low cost, and eco-friendly activation scheme for producing highly efficient porous carbon from different types of lignocellulosic biomass for phenol and methylene blue dye removal. To the best of our knowledge, there is no study showed the comparison between physical and chemical activation on the surface texture properties of AC derived from different lignocellulosic wastes for organic pollutants adsorption.

Herein, sugarcane bagasse and sawdust were used as lignocellulosic biomass waste precursors to develop of carbon-based materials. Afterwards, the physical and chemical activation schemes were applied to improve surface texture and properties. The as-prepared AC samples were characterized via various techniques, including N_2_-Physisorption, X-ray diffraction (XRD), X-ray photoelectron spectroscopy (XPS), Thermogravimetric analysis (TGA), Fourier transform infrared (FTIR), and scanning electron microscopy (SEM). Their activity and recyclability toward phenol removal from an aqueous solution were evaluated through optimizing adsorption conditions. Finally, adsorption kinetics and isotherms were studied to investigate the best models that better describe the adsorption process.

## Experimental section

### Chemicals

All chemicals used in this study were analytical grade and used without further purification, including potassium carbonate (K_2_CO_3_), potassium hydroxide (KOH), sulfuric acid (H_2_SO_4_), and nitric acid (HNO_3_).

### Characterization techniques

TGA curve of the precursors were recorded up to 1000 °C in nitrogen atmosphere using the TA 60 thermal analyzer apparatus (Shimadzu, Japan) where a heating rate of 10 °C min^−1^ was applied. XRD patterns of the samples were carried out on Bruker D8 Advance using Cu Kα radiation at a wavelength of 1.5406 Å. FTIR spectra were recorded using Shimadzu-470 in the wavenumber range of 4000–400 cm^−1^ using the KBr disc technique. The sample micro-morphologies were examined using SEM (JSM-6360LA). A Thermo Fisher (K-alpha) instrument of monochromated micro-focused Al Kα radiation (1486.6 eV) was used to obtain XPS measurements. N_2_ adsorption–desorption isotherm (Quantachrome Instrument Corporation, Nova 3200, USA) was employed to determine the specific surface areas by using Brunauer–Emmett–Teller (BET) calculations and the pore size distributions were recorded by desorption branch using BJH method and t-method.

### Pre-treatment and analysis of raw materials

In order to use raw material as a lignocellulose precursor for producing porous carbon, the sugarcane bagasse was pre-treated to remove impurities. The lignocellulose precursors employed in this study, sugarcane bagasse (BS) and sawdust (SD), were washed with hot distilled water to remove the inorganic impurities, then dried at 100 °C for 24 h to remove water. The dried sugarcane bagasse was grinded using a 4-blade mechanical grinder (Moulinex, model: E108 CHB, 500 W) and sieved with a 2 mm mesh. The sieved bagasse was separated from the un-sieved one, therefore we obtained three main sources of lignocellulosic materials namely, bagasse un-sieved (BU), bagasse sieved (BS) and sawdust (SD) as indicated in Fig. S1.

After pre-treatment, the composition contents (Cellulose, Hemi-Cellulose and Lignin) of the products were analyzed using National Renewable Energy Laboratory analytical procedures^28^. Briefly, 1.0 g of sample “s” was added to 150 mL distilled water, then refluxed for 1 h at 90–95 °C. The produced weight “a” was mixed with 150 mL sulfuric acid (1 M), then refluxed for 1 h. Subsequently, the produced weight “b” was kept in 10 mL sulfuric acid (72%) for 4 h followed by adding 150 mL sulfuric acid (1 M), then refluxed for 1 h and the produced weight was “c”. The quantitative analysis of the samples was determined according to the following equations:

1 $$\mathrm{Percentage \,of\, hemicellouse}=\frac{\mathrm{a}-\mathrm{b}}{\mathrm{s}}\times 100$$

2 $$\mathrm{Percentage\, of\, cellouse}=\frac{\mathrm{b}-\mathrm{c}}{\mathrm{s}}\times 100$$

3 $$\mathrm{Percentage \,of\, lignin}=\frac{\mathrm{c}}{\mathrm{s}}\times 100$$

In order to determine the moisture content of the as-treated samples, the thermal drying method was applied. Typically, 1.0 g of start materials were placed in a pre-weighted, clean and dried crucible and weighed. After that, the crucible was heated gently in an air-dry furnace to 105 °C in and kept for 4 h. Subsequently, the sample was cooled to ambient temperature and weighed. The moisture percentage (M) is estimated from the difference between initial and final weight according to the following equation, Eq. (4):

4 $$\mathrm{M}=\frac{{\mathrm{W}}_{2}-{\mathrm{W}}_{3}}{{\mathrm{W}}_{2}-{\mathrm{W}}_{1}}\times 100$$

where M (%), W_1_ (g), W_2_ (g), and W_3_ (g) refer to the percentage moisture content, the weight of the empty crucible, the weight of the sample and crucible before drying, and the weight of the dried sample and the crucible, respectively. For volatile matter determination, 1.0 g of the BU, BS, SD powdered samples were put in a ceramic crucible and heated in a muffle furnace at 900 °C for 7 min, and then cooled to room temperature. The volatile matter was calculated using the following equation, Eq. (5):

5 $$\mathrm{VC}=\frac{{\mathrm{w}}_{2}-{\mathrm{w}}_{3}}{\mathrm{w}}\times 100-\mathrm{M}$$

where VC (%), w (g), w_2_ (g), and w_3_ (g) refer to the percentage volatile matter content, the weight of the sample before heating, the weight of the sample and crucible before drying, and the weight of the sample and the crucible after heating, respectively. The procedure for ash content determination is similar to volatile matter determination except that the furnace temperature was kept at 900 °C for 2 h. The ash content was calculated using the following equation, Eq. (6):

6 $$\mathrm{AC}=\frac{{\mathrm{w}}_{4}}{\mathrm{w}}\times 100$$

where AC (%) and w_4_ (g) refer to the percentage ash content and the weight of heated sample, respectively. Finally, the fixed carbon is determined via the following equation:

7 $$\mathrm{FC}=100-(\mathrm{VC}+\mathrm{AC})$$

where FC (%) and w_4_ (g) refer to the percentage fixed carbon of the sample.

### Preparation of porous carbon materials

The thermal treatment of raw materials used for preparing carbon products and schematic diagram of the experimental setup of pyrolysis test is shown in Fig. S2a. Typically, the dried samples of approximately 10 g were placed in a ceramic crucible. The sample was heated at a heating rate of 3 °C min^−1^ until 300 °C and maintained for 1 h then the temperature was raised by the same heating rate up to 750 °C and maintained for 2 h. The samples were then cooled naturally to ambient temperature. The yield of the produced carbon materials was ranged from (20–23%) and these carbonization products of raw materials were donated as BU_car, BS_car, and SD_car. Following the carbonization step, activation step was performed. There are two general methods applied for the activation process, either physically or chemically. Physical activation is conducted in presence of water vapor as the activator, while we have chosen both KOH and K_2_CO_3_ as the main chemical activators in this study.

#### Physical activation via water vapor

Physical activation achieved by steam in a tubular furnace at different temperatures ranged from 700 to 950 °C as shown in Fig. S2b. Samples were placed in an alumina boat like crucible and heated at heating rate of 5 °C min^−1^, then maintained for 2 h at the target temperature then cooled naturally to ambient temperature. Samples were continuously purged with Ar gas as a carrier gas saturated with steam (90–100 °C) at a flow rate of 50 mL min^−1^ and these samples were donated as BU_AC_H_2_O_X where X is the activation temperature.

#### Chemical activation using KOH and K_2_CO_3_

In chemical activation using K_2_CO_3_ and KOH, we have studied two parameters, activation temperature and amount of activator. Firstly, with fixed ratio between the carbon samples and the activating agent of 1:1 (w/w), samples were first weighed and uniformly mixed with the activating agent with aid of a small amount of distilled water then dried. After that they were placed in alumina boat crucible inside a tube furnace. Then samples were purged for 15 min with Ar gas at 50 mL min^−1^ flow rate to replace the air inside the quartz tube. Then the samples were heated to the target temperature (700, 750 or 800 °C) at heating rate of 5 °C min^−1^ and maintained at the target temperature for 2 h, then cooled naturally to room temperature and these samples were donated as BU_AC_KOH_X and BU_AC_K_2_CO_3__X where X is the activation temperature. Second, the mass ratio of carbon samples to activating agent was controlled at 1:1, 1:2 or 1:3 (w/w) at fixed temperature of 700 °C as it gives the highest yield. Samples were first weighed and uniformly mixed with the activating agent with aid of a small amount of distilled water then dried. After that the samples were heated with the same sequence as abovementioned. Finally, the activated carbon product was washed with hydrochloric acid then distilled water till the pH became neutral and was dried at 100 °C overnight for further analysis.

### Adsorption tests

The adsorption efficiency of the as-prepared samples for phenol removal was assessed via dispersing 50 mg of the porous carbon materials into 100 mg L^−1^ of phenol solution under controlled temperature. The suspension was put inside an incubator orbital shaker (JSR, JSSI-100C), while samples were withdrawn at different time intervals. Subsequently, each sample was filtrated first, and their absorbance was measured using UV–Vis spectrophotometer (PERKIN ELMER, Lambda 40). Absorbance of samples at 269 nm was acquired and used to measure the concentration using the calibration curve constructed based on Beer’s-Lambert law as shown in Fig. S3. For adjusting the adsorption conditions, the effect of different parameters was studied, initial phenol concentration (50–250 ppm), solution pH (2–10) using 1 M solution of NaOH or HCl, contact time (5–60 min), and temperature (25–45 °C). After that, the optimum conditions were chosen for the next adsorption tests. The removal efficiency of the carbon sample was calculated using the following empirical formula, Eq. (8):

8 $$\mathrm{Removal \,efficiency}=\frac{{\mathrm{C}}_{0}-{\mathrm{C}}_{\mathrm{e}}}{{\mathrm{C}}_{0}}\times 100$$

where C_0_ and C_e_ are the initial and equilibrium concentrations of phenol, respectively. Also, the maximum adsorption capacity was determined by the following equation, Eq. (9):

9 $$\mathrm{Adsorption \,capacity}=\left({\mathrm{C}}_{0}-{\mathrm{C}}_{\mathrm{e}}\right)\times \frac{\mathrm{V}}{\mathrm{m}}$$

where V and m are volume of solution and mass of carbon sample, respectively. For methylene blue adsorption experiments, a typical procedure is followed similar to that of phenol test without pH adjustment. Absorbance of samples at 664 nm was acquired and used to measure the concentration using the calibration curve constructed based on Beer’s–Lambert law as shown in Fig. S4.

## Results and discussion

### Structural and morphological characterization

The chemical composition of raw materials-derived carbons has an effect that cannot be neglected on the adsorption behavior of activated carbon materials^29^. Accordingly, lignocellulosic raw materials (BU, BS, and SD) were analyzed to assess their content of cellulose, hemicellulose, lignin, moisture, volatile content and ash content. Results of all samples were comparable as shown in Table S1. The content of lignin in the sawdust sample was almost double that was found in the sugarcane bagasse sample. The high content of lignin could effectively increase the formation of microporous structure of AC, in contrast the cellulose which may lead to mesoporous formation^30^. The carbon yield of all samples after the pyrolysis step was in the range of 19–24%. Furthermore, TGA was also performed to evaluate the volatile, ash, and fixed carbon content as indicated in Fig. S5. In general, thermal degradation of lignocellulosic materials consists of three main steps, dehydration in the first step, cellulose and hemicellulose decomposition in the second step, and degradation of lignin takes place in the final step. Consequently, it can be observed from TGA curves that the first loss at less than 200 °C corresponds to the moisture content which represents 8, 12, and 6% for BU, BS and, SD, respectively. The second loss at a temperature less than 400 °C accounts for more than 60% of the total sample weight. The final weight loss takes place at a temperature of 500 °C and remains constant till it reaches 1000 °C whereas only fixed carbon remains, which accounts for about 6%. The residual amount of carbon observed from TGA is much less than the actual yield of the carbonization process. This could be due to the low purity of N_2_ gas (99%) used and the very low amount of sample (> 5 mg) used in TGA.

The amount of carbon generated from the activation process is a very significant factor because it has to do with the application on a large scale. Consequently, the yield of activated carbon materials can be calculated by applying the following equation:

10 $$\mathrm{Yield }\left(\mathrm{\%}\right)=\frac{\mathrm{mass\, of \,AC \,produced}}{\mathrm{initial\, mass \,of \,sample}}\times 100$$

**Figure 1:**
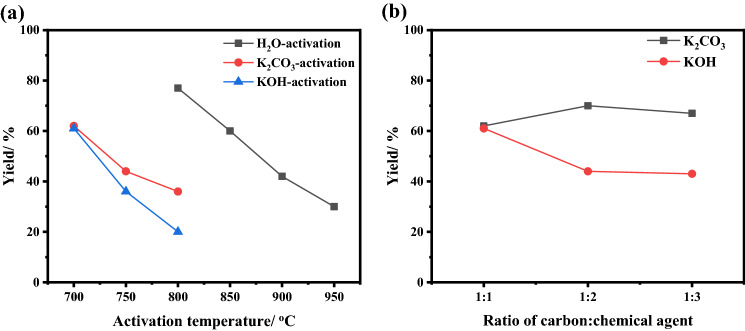
The calculated yield of activated carbon with various activators, water vapor, K_2_CO_3_, and KOH, at different temperatures (**a)** and ratio of chemical agents (**b**) of BU_car sample.

The yield of AC as a function of the activation temperature and different activators is presented in Fig. 1a. Generally, the increase in the activation temperature sharply decreased the carbon yield, this could be attributed to more volatiles being eliminated, that is, cellulose and hemicellulose^31^. For water-, K_2_CO_3_-, and KOH-activation, the yield decreased from 77% at 800 °C to 30% at 950 °C, 62% at 700 °C to 36% at 800 °C, and 61% at 700 °C to 20% at 800 °C, respectively. The weight effect of chemical activating agents on the yield were also assessed as shown in Fig. 1b. A slight increase in the yield of carbon was observed when increasing the amount of potassium carbonate, while increasing the amount of potassium hydroxide significantly decreased the yield. This can be explained based on that the proposed chemical reaction occurring between carbon and KOH leads to the release of CO, CO_2_, H_2_ gases, while K_2_CO_3_ could be reduced by carbon to metallic potassium during activation process^32^. The obtained results are in line with those previously reported^20,33^.

$$\begin{aligned} & {\text{K}}_{{2}} {\text{CO}}_{{3}} + {\text{3C}} \to {\text{2K}} + {\text{3CO}} \\ & {\text{6KOH}} + {\text{2C}} \to {\text{2K}} + {\text{2K}}_{{2}} {\text{CO}}_{{3}} + {\text{3H}}_{{2}} \\ & {\text{K}}_{{2}} {\text{CO}}_{{3}} \to {\text{K}}_{{2}} {\text{O}} + {\text{CO}}_{{2}} \\ & {\text{2K}} + {\text{CO}}_{{2}} \to {\text{K}}_{{2}} {\text{O}} + {\text{CO}} \\ \end{aligned}$$

During the activation processes, whether chemically or physically, lignocellulosic materials disintegrate through two stages: degradation of cellulose and hemicellulose, which leads to the formation of pores on the carbon surface and promotes the dispersal of the oxidizing agent, followed by the interaction of lignin with the oxidizing agent to produce AC. It can be concluded that the removal of volatile materials and the reaction that occurred between sample and activator are the main reasons for the reduction of AC yield.

**Figure 2:**
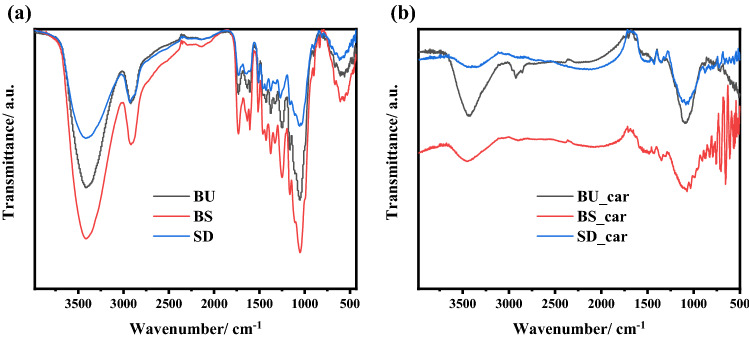
FTIR spectra of (**a**) BU, BS, and SD raw materials and (**b**) BU_car, BS_car, and SD_car carbonized products.

To investigate functional groups, the FTIR spectra of BU, BS, and SD raw materials and their carbonized products were carried out and the results were shown in Fig. 2. One can see that the spectra obtained from raw lignocellulosic materials have many similar features as shown in Fig. 2a. An O–H group results in a wide band located in the range of 3200–3600 cm^−1^.^34^, while C–H symmetric and asymmetric vibration are presented by the band at 2922 cm^−1^^35^. The two bands centered at 1516 and 1424 cm^−1^ are assigned to skeletal C=C vibrations of aromatic rings and the band at 1629 cm^−1^ may be attributed to the stretch vibrations of C=C bonds in olefinic structures^34^. C–O vibrations are probably the cause of the adsorption present between 1000 and 1350 cm^−1^. Most of the absorption bands disappeared after complete carbonization (Fig. 2b) processes, indicating the organic matters vaporization leading to formation of intrinsic micropores.

**Figure 3:**
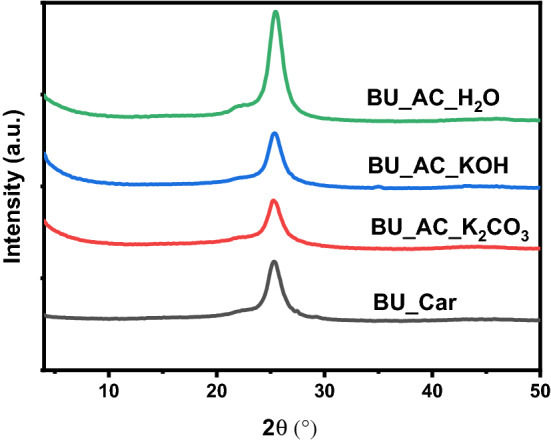
XRD patterns of BU_car, BU_AC_H_2_O, BU_AC_K_2_CO_3_, and, BU_AC_KOH.

XRD patterns of carbonized BU, BU_AC_H_2_O, BU_AC_K_2_CO_3_, and, BU_AC_KOH were recorded to investigate their crystallinity and the results are presented in Fig. 3. As shown, the carbonized BU product exhibited main diffraction peak located at 25.4° assigned to (002) plane of disordered carbon structures. The existence of broad peaks with relative low intensities at these 2θ implies the amorphous nature and low graphitization of material. After the activation process using different activators, the amorphous nature of carbon material did not greatly change. However, the peaks became more defined than un-activated carbon demonstrating that the activation processes are useful for the construction of a disorderly carbon structure in the activated carbon. What’s more is that no diffraction peaks corresponding to KOH and K_2_CO_3_ crystals were observed, implying that washing could completely remove the impurities existing on the surface of activated carbon.

**Figure 4:**
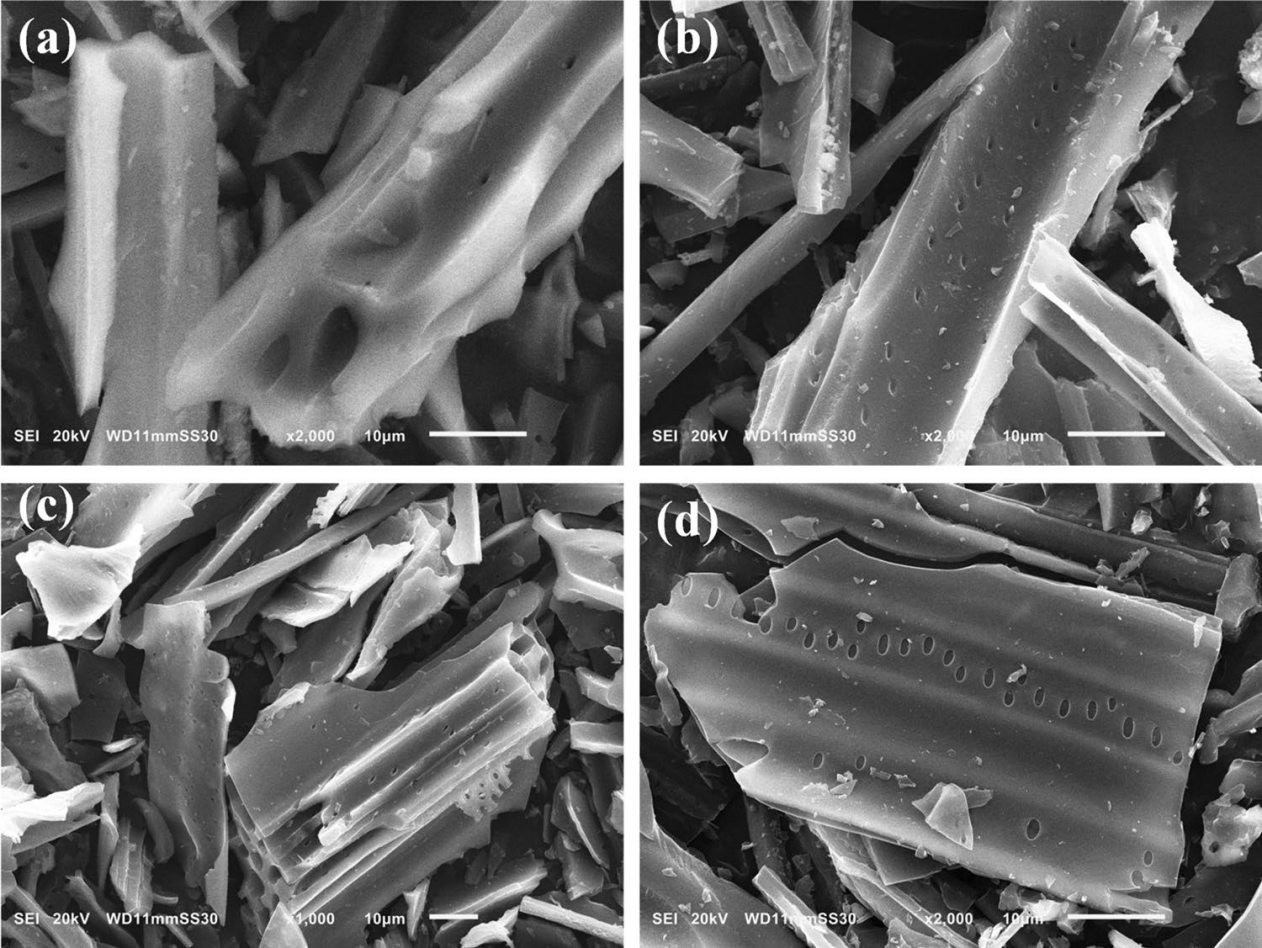
SEM images of (**a**) BU_car, (**b**) BU_AC_H_2_O, (**c**) BU_AC_K_2_CO_3_, and, (**d**) BU_AC_KOH.

The surface texture and micro-morphological structure of carbonized BU, BU_AC_H_2_O, BU_AC_K_2_CO_3_, and, BU_AC_KOH, were examined by SEM imaging as shown in Fig. 4. One can observe the tube bundle structure of carbonized BU possessing many pores which is favorable for harmful organic pollutants adsorption. The activated carbon materials showed the same microstructure, tube bundle, as displayed in Fig. 4b–d. Nevertheless, the porosity is enriched significantly and also the interconnected pores in the activated carbon can promote the dyes and phenols adsorption onto the adsorbent surface.

**Figure 5:**
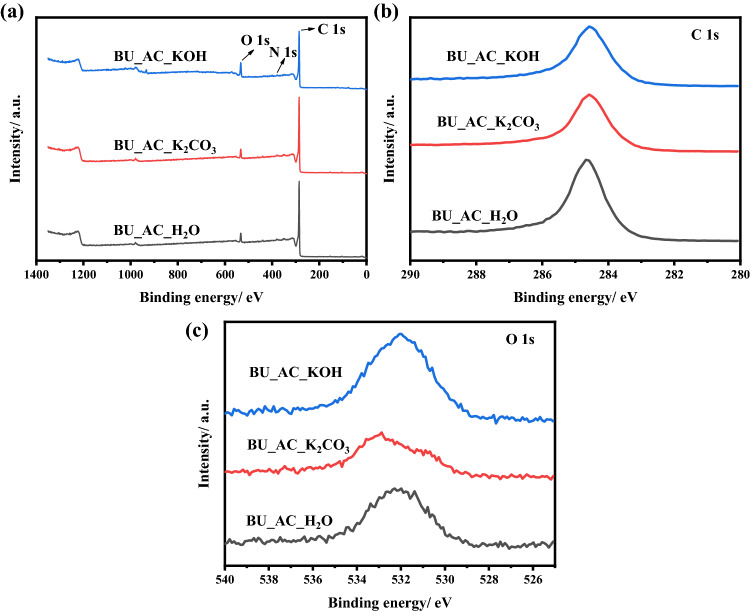
XPS survey spectra (**a**), high-resolution XPS spectra of C 1 s (**b**), and O 1 s (**c**) of BU_AC_H_2_O, BU_AC_K_2_CO_3_, and BU_AC_KOH porous materials.

Surface composition analysis of various activated carbon samples was studied through X-ray photoelectron spectroscopy (XPS) and the results are displayed in Fig. 5. All the activated samples showed three main peaks centered at 285, 395, and 532 eV which are corresponds to C 1 s, N 1 s, and O 1 s, respectively^36–38^. The surface elemental analysis of all the samples (Table 1) are almost the same except that the BU_AC_KOH exhibited a relatively higher oxygen content. Accordingly, the activation process using KOH-activator can increase the oxygen-containing functional groups on the surface of the activated carbon resulting in promotion of organic pollutants removal. The oxygenated function-groups that present on the surface of adsorbents give them negative polarity, this leads to electrostatic attraction between the adsorbent and MB via electrostatic mechanism^39^. In case of phenol molecules, the surface of carbon could act as an electron donor due to the presence of oxygen containing function groups on the surface of the AC, while the aromatic phenol ring can act as an electron acceptor, this induce electron donor–acceptor mechanism formation.

**Table 1:** Surface atomic ratio and compositions of activated carbon samples.

Sample	Elemental analysis (%)			Element ratio		
	C	O	N	O/C	N/C	(O + N)/C
BU_AC_H_2_O	94.21	4.56	0.28	0.048	0.003	0.051
BU_AC_K_2_CO_3_	93.68	5.00	0.55	0.053	0.006	0.059
BU_AC_KOH	85.69	8.75	0.85	0.102	0.009	0.112

**Figure 6:**
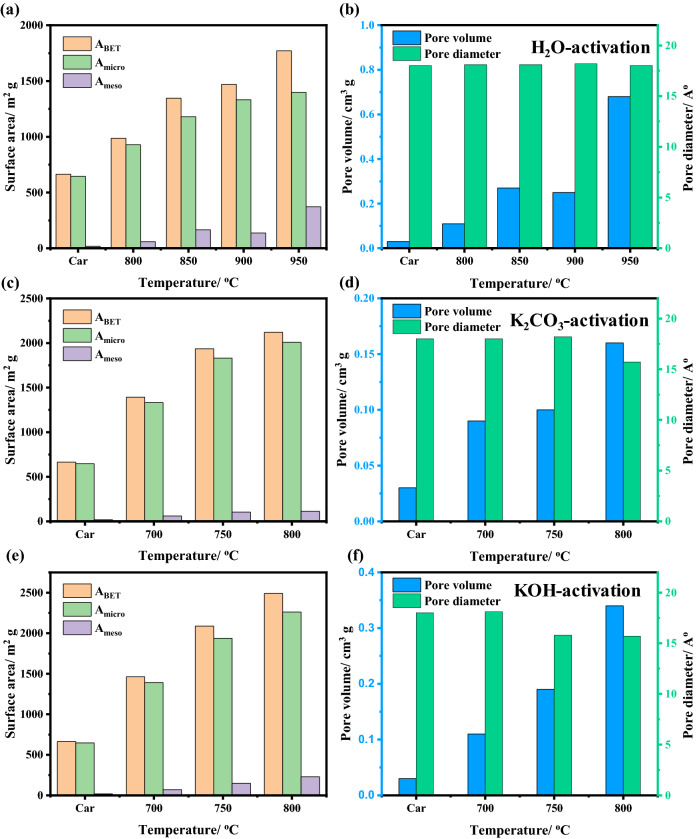
Specific surface areas, pore volume, and pore diameter of BU-car sample activated with various activators, water vapor (**a**, **b**), K_2_CO_3_ (**c**, **d**), and KOH (**e**, **f**), at different temperatures.

The activation temperature and weight of the chemical activators have an important role in creating pores over carbon materials. In general, the process of pore formation consists of three basic steps: exposing previously inaccessible pores, new pores formation through selective activation, and expansion of pre-existing pores. So, specific surface area, surface texture, and pore size distribution were acquired via N_2_-physisorption technique^40^ and the results are summarized in Table S2. It is worth mentioning that the un-sieved bagasse sample was used to optimize the preparation conditions using various activators (water vapor, K_2_CO_3_ and KOH) as shown in Fig. 6. After that both the sieved bagasse and sawdust carbonized samples were treated under the optimized conditions obtained previously. As mentioned above the raw materials were carbonized at a temperature of 750 °C, the resulting surface area was about 664 m^2^ g^−1^. The activation temperature effect on surface texture properties was evaluated by varying the range of temperature from 800 to 950 °C and from 700 to 800 °C in case of physical and chemical activation, respectively. Increasing the temperature increases the surface area (S_BET_), micropore volume and total pore volume. The S_BET_ values obtained for water-, K_2_CO_3_-, and KOH-activation were 1771 m^2^ g^−1^ at 950 °C, 2120 m^2^ g^−1^ at 800 °C, and 2490 m^2^ g^−1^ at 800 °C, respectively. Similar trends were observed for the total pore volume and micropore volume obtained from the BJH plot and t-plot. This indicated that an increase in temperature resulted in an increase of the surface area stimulating the generation and formation of new micropores. This is because higher temperatures promote the crack of the hydrocarbon bonds in, lignocellulose, the elimination of hydroxyl and carboxyl groups, and the creation of the porous material of the carbon-based adsorbents. Notably, the weight ratio between carbon sample and chemical activators are fixed to be 1:1. Although the significant surface area values of AC at were obtained at higher temperatures, the yield was found to be quite low (Fig. 1), which in turn reduces its wide applicability. Therefore, physical activation at 900 °C and chemical activation at 700 °C was supposed to be optimal conditions because of their reasonable surface area and high yield.

**Figure 7:**
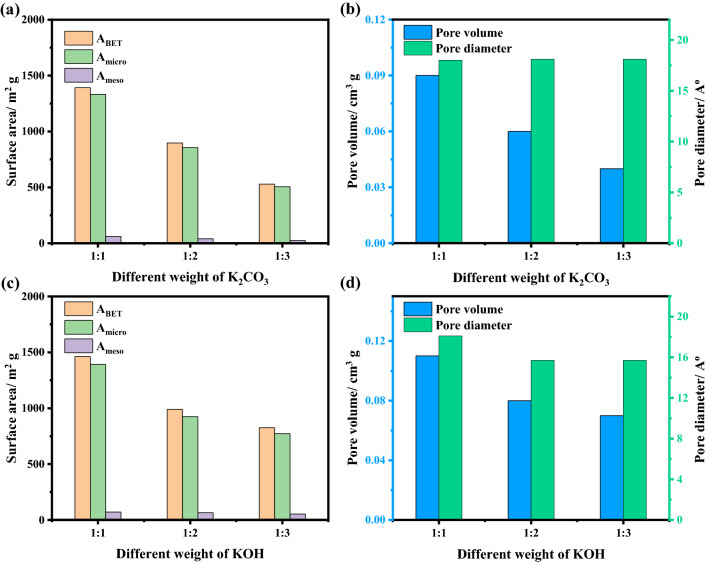
Specific surface areas, pore volume, and pore diameter of BU-car sample activated with weight ratio of activators, K_2_CO_3_ (**a**, **b**), and KOH (**c**, **d**).

After that, the weight effect of chemical activators, K_2_CO_3_ and KOH, on activation process was studied at a constant activation temperature of 700 °C as shown in Fig. 7. Unlike the activation temperature, increasing the amount of the K_2_CO_3_ or KOH mass ratio from 1 to 3 resulted in a significant decrease of the specific surface area and total pore volume (Table S2). This is because the high concentration of the activating chemical over-reacts with the carbon structure resulting in the destruction of the pores. This also could be explained in the light of the major part of specific surface area is mostly produced via micropores as indicated by the results obtained by the t-method.

**Figure 8:**
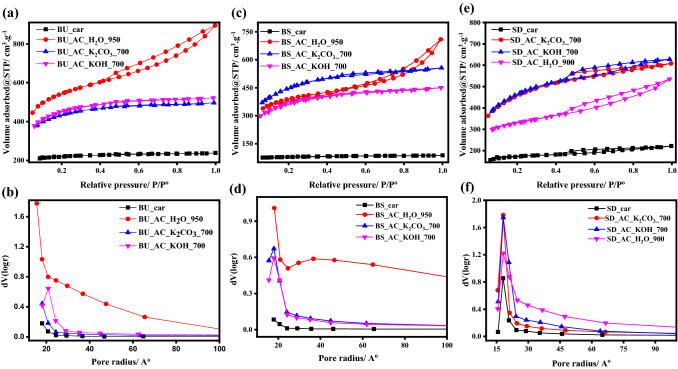
The N_2_ adsorption–desorption isotherm and particles pore distribution of BU (**a**, **b**), BS (**c**, **d**), and SD (**e**, **f**) and their activated products using water vapor, K_2_CO_3_, and KOH-activators.

After obtaining the optimized conditions for physical and chemical activation processes, these conditions were applied to sieved bagasse and sawdust carbonized samples and the results are tabulated in Table 2. The surface texture properties, principally surface area and pore volume were obviously enhanced after activation either physically or chemically compared with the carbonized samples. Figure 8 shows the N_2_ adsorption–desorption isotherm and pore-size distribution of different BU, BS, SD-carbon samples. As it can be seen, all the obtained isotherms of carbon samples are of Type I. Microporous materials, those with pore diameters less than 2 nm, give this type of isotherm indicating that the activated carbon contains major microporous structure and a minimal amount of mesoporous structure. The varying responses of raw materials can be explained based on the chemical compositions of lignocellulosic wastes applied in terms of the hemicelluloses, cellulose, and lignin content inside the raw materials as shown in Table S1. In general, both the cellulose and lignin content are higher in case of the un-sieved sugarcane bagasse, while in case of the sieved sample, the hemicellulose content was higher. During the carbonization of lignocellulosic materials, hemicellulose is first decomposed, followed by cellulose, and finally lignin is broken down^29^. The difference in chemical constitutes of the raw materials leads to formation of porous carbon with various surface texture properties.

**Table 2:** Surface texture properties BS and SD-carbon samples activated with different activators under optimized conditions.

Sample	BET method		t method		BJH method	
	Surface area (m^2^ g^−1^)	Micropore volume (cm^3^ g^−1^)	Micropore area (m^2^ g^−1^)	External area (m^2^ g^−1^)	Pore volume (cm^3^ g^−1^)	Pore radius (A^o^)
BS_car	243	0.12	235	8	0.01	17.9
BS_AC_H_2_O_900	1239	0.47	905	334	0.60	18.1
BS_AC_K_2_CO_3__700	1482	0.69	1374	107	0.17	17.9
BS_AC_KOH_700	1204	0.57	1120	84	0.14	17.9
SD_car	534	0.25	479	55	0.11	18.1
SD_AC_H_2_O_900	1053	0.43	819	233	0.41	18.1
SD_AC_K_2_CO_3__700	1528	0.70	1373	251	0.27	18.1
SD_AC_KOH_700	1544	0.69	1360	184	0.31	18.2

**Figure 9:**
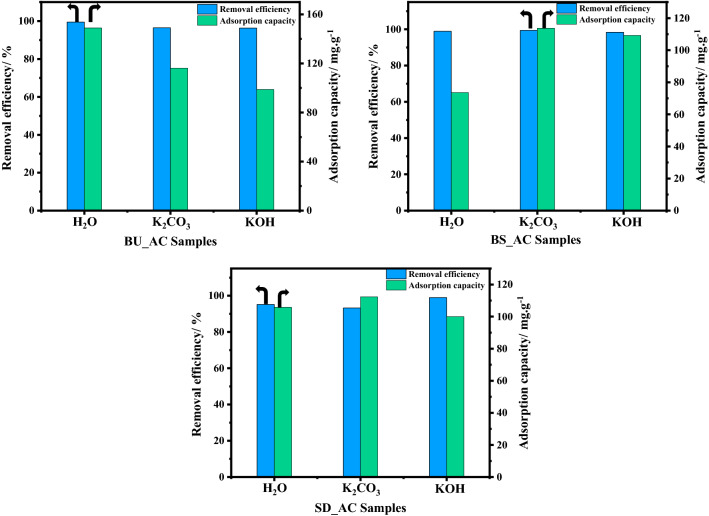
Maximum adsorption capacity and adsorption efficiency of MB solution under the same conditions of temperature, pH, and MB dose (25 °C, 6.5–7, 100 mg L^−1^) for AC samples.

Water-soluble methylene blue (MB, C_16_H_18_ClN_3_S, M. wt of 319.86 g mol^−1^) is one of the most common pollutants that are used to evaluate the removal capacity of porous carbon materials and others. The adsorption capacity of MB could be an indication of the mesoporous nature because its molecule size is higher than 2 nm. Consequently, the activated carbon materials were mixed with MB solution under the same conditions of temperature, pH, contact time, and MB dose (25 °C, 6.5–7, 60 min, 100 ppm), and the results were presented in Fig. 9. It is worth noting that most of the MB concentration was removed within the first 5 min (Fig. S6), indicating that the samples possess a high degree of porosity. Moreover, all samples have shown high performance toward MB removal, with a removal efficiency exceeding 90% as shown in Fig. 9. Among all samples, the carbonized BU sample activated via water vapor showed the highest adsorption capacity of 148.8 mg g^−1^ with very fast dye removal. This could be explained due to water vapor creating micro/meso pores over the carbon materials resulting in MB molecules being efficiently adsorbed onto the adsorbent surface. On the other side, the pyrolyzed BS and SD samples activated via chemical activators showed a comparable removal efficiency with a gradual increase in the adsorption along the contact time. The obtained results from MB adsorption are consistent with those from BET surface area, indicating that the applied preparation schemes efficiently transform lignocellulosic waste into highly efficient porous carbon materials.

### Adsorption of phenol

As highly porous materials, the as-prepared activated carbon samples can be used for the removal of organic pollutants (e.g., methylene blue, phenol) in aqueous solutions. Generally, the adsorption process was performed by adjusting some parameters, mainly including initial concentration, contact time, temperature and pH value. So, to obtain highly efficient carbon as an adsorbent for phenol removal, a detailed study of adsorption conditions was studied as follows:

#### Effect of initial concentration

**Figure 10:**
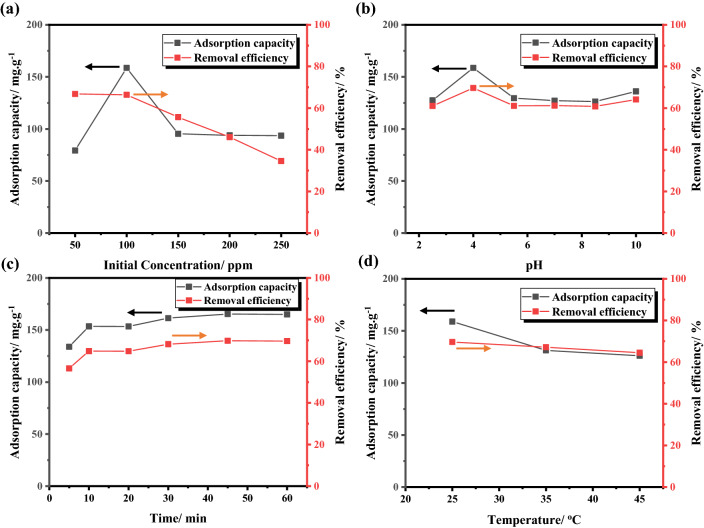
(**a**) Initial concentration (temperature of 25 °C, pH of 4–4.5), (**b**) pH (phenol dose of 100 mg L^−1^, temperature of 25 °C), (**c**) Contact time (phenol dose of 100 mg L^−1^, temperature of 25 °C, pH of 4), and (**d**) Temperature effect (phenol dose of 100 mg L^−1^, pH of 4) on adsorption of phenol over SD_AC_H_2_O_900 sample.

The concentration effect of adsorbate on adsorption process was evaluated by varying the phenol dose in the range of 50–250 mg L^−1^ at 25 °C. As shown in Fig. 10a, the removal efficiency decreased obviously with increasing the initial concentration of phenol. This can be discussed as; at first, the quantity of adsorbent molecules was higher than that of adsorbate molecules, resulting in an efficient surface area available for adsorption^41^. Accordingly, the highest adsorption efficiency (approximately ~ 66%) was obtained at low concentration in the range of 50–100 ppm. Furthermore, exceeding the phenol concentration to the optimum dosage amount would cause a drastic drop in the removal efficiency. Such reduced removal was because the number of adsorbent molecules was less than that of adsorbate molecules, providing a low surface area for adsorption which results in decreasing the removal percentage. It can be observed that the relation between adsorption capacity and initial concentration showed a little change than removal efficiency (Fig. 10a). Particularly, the maximum adsorption capacity of 158.9 mg g^−1^ was noted at concentration of 100 ppm. Therefore, this initial concentration, 100 mg L^−1^ of phenol, was chosen for the subsequent adsorption experiments.

#### Effect of pH

Figure 10b shows the phenol removal efficiency and its corresponding adsorption capacity at pH 2–10, while the initial concentration and temperature are constant at 100 mg L^−1^ and 25 °C. The adsorption of phenolic compounds on activated carbon materials is essentially dependent on the pH of the solution, attributed to the pH greatly influencing the ionization degree and surface charge of porous carbon. As agreed, the zero-point charge of carbon is ~ 8, meaning that the surface charge will be positive below this pH. Also, the dissolution constant (pKa) of phenol is approximately 10, indicating that the phenol molecules will be in an un-ionized state underneath this pH. As shown, the phenol removal and adsorption capacity were significantly increased by increasing the solution pH from 2 to 4. This can be explained as, at pH = 2, adsorbate molecules will combine with the positively charged surface of carbon through electrostatic attraction. When the pH was increased, the adsorbate molecules will slightly transfer to ionized state and the adsorbent effectively attracts the phenoxide ion. On a particular note, the optimal pH for phenol adsorption was 4.0, which lines with previous reports^41^. In spite of this, greater than pH 4.0, the removal efficiency decreased because the negatively charged surface of carbon will repel phenoxide ions. At pH = 10, the surface of activated carbon and phenol molecules were negatively charged resulting in increasing electrostatic repulsion. Thus, the solution pH was adjusted to be 4 for the subsequent adsorption experiments.

#### Effect of contact time

To determine the required time for the equilibrium process between adsorbent and adsorbate, the effect of contact time on adsorption efficiency is provided in Fig. 10c. As shown, the adsorption efficiency of phenol increases as the time increase till reaching equilibrium within 10 min, after which there is a plateau nature demonstrating that the equilibrium is attained. This could be attributed to the vacant sites for adsorption being available and large, thus efficient electrostatic attraction occurred between bulk-carbon and solute phase^42^. With increasing time, the competition between the phenol molecules increases to occupy the rarely available active sites which in turn weakens the driving force between phenol and as-prepared carbon.

#### Effect of temperature

Figure 10d shows the effect of the operating temperature on removal of phenol over the prepared carbon sample in the range from 25 to 45 °C, while the initial concentration and solution pH are constant at 100 mg L^−1^ and 4, respectively. As the temperature is elevated, the removal efficiency decreased suggesting that the removal process of phenol over as-prepared carbon is spontaneous and exothermic in nature. The removal efficiency percent of the sample was ~ 66% at 25 °C, with an adsorption capacity of 158.9 mg g^−1^. The reason behind the decrease in adsorption efficiency as temperature increases is because of the weakened physical bond between the adsorbate and adsorbent which caused the partial removal of phenol molecules from porous carbon structure^43^. Accordingly, the operating temperature was fixed at 25 °C for the subsequent adsorption experiments.

It can be noted that both the removal efficiency and the absorption capacity took almost the same trend, as there was a large and rapid increase observed at the beginning and then a very slight increase before the equilibrium process occurred later. This was clearly due to the availability of adsorption active sites at the beginning, however, later on, the remaining sites are affected by the repulsive forces present between the molecules on the carbon surface. Therefore, these optimized parameters were chosen for the subsequent adsorption experiments.

**Figure 11:**
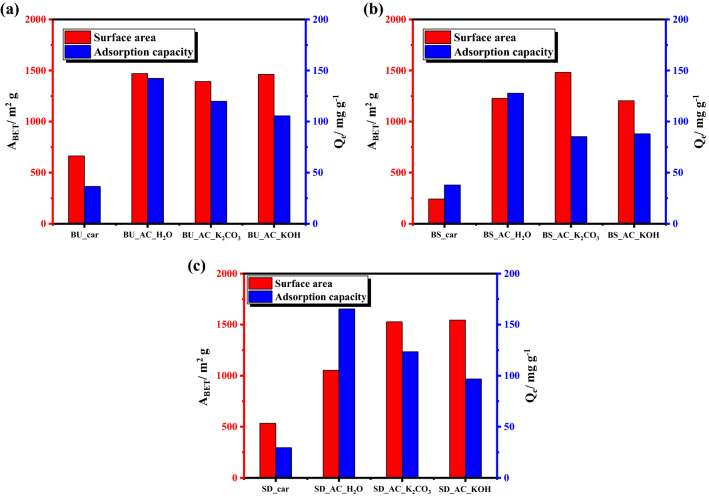
Specific surface area and adsorption capacity for phenol removal of BU (**a**), BS (**b**), and SD (**c**) samples activated via physical and chemical activation processes under adsorption conditions of phenol dose of 100 mg L^−1^, temperature of 25 °C, and pH of 4.

After selecting the optimum conditions for phenol removal over porous carbon, we applied these conditions to other as-prepared samples for evaluating the activation processes (chemical and physical) as shown in Fig. 11 and the results are tabulated in Table S3. It is generally accepted that the removal efficiency of adsorbate depends on the specific surface area of the adsorbent. Although, the maximum adsorption property was obtained for the SD_AC_H_2_O_900 sample, the surface area was 1053.0 m^2^ g. This shows the pivotal role of the surface functionalization of carbon via water activation for phenol chemisorption compared with chemical activation, namely, KOH and K_2_CO_3_. The adsorption capacity of the fabricated SD_AC_H_2_O_900 sample is 165.3 mg g^−1^ with phenol adsorption efficiency of 69.7%. Compared to carbonized SD sample, the adsorption efficiency was significantly enhanced; attributed to the activator role in creation of pores for adsorption processes. The yield of the porous carbon materials is crucial and a determining parameter for large scale application in terms of cost and economic value. The carbon products from sawdust and bagasse un-sieved exhibited a high percentage of carbon yield compared with bagasse sieved ~ 60–67%. This can be understood in the light of lignin content in raw materials, which is regarded as carbon source. The maximum adsorption capacities of Phenol onto as-prepared AC in this study and in the literature are summarized in Table 3.

**Table 3:** Comparison between SD_AC_H_2_O and other activated carbons used for phenol adsorption.

Precursor material	Activator	pH	T (°C)	Q_e_ (mg g^−1^)	Refs.
Sugarcane bagasse	H_2_O	4.0	25	159	This study
Black wattle bark waste	ZnCl_2_	6.5	55	99	^13^
Pyrolytic tyre	HNO_3_	6.7	25	52	^44^
Sewage sludge	HNO_3_	6.8	20	30	^45^
Waste tea	HNO_3_	7.0	30	108	^46^
Fox nutshell	ZnCl_2_	7.0	30	77	^47^
Peanut shell	–	6.0	–	21	^48^
Coconut shell	–	–	25	145	^49^
Avocado kernel seed	CO_2_	6.0	25	90	^50^
Rattan sawdust	–	–	30	149	^51^

#### Adsorption kinetics

In this study, different kinetics models were employed to describe the adsorption mechanism of phenol molecules over activated carbon samples, namely, pseudo-first order and pseudo-second order. The mathematical expressions of pseudo-first order (Eq. 11) and pseudo-second order (Eq. 11) as follows:

11 $$\mathrm{ln}\left({\mathrm{Q}}_{\mathrm{e}}-{\mathrm{Q}}_{\mathrm{t}}\right)={\mathrm{lnQ}}_{\mathrm{e}}-{\mathrm{k}}_{1}\mathrm{t}$$

12 $$\frac{1}{{\mathrm{Q}}_{\mathrm{e}}-{\mathrm{Q}}_{\mathrm{t}}}=\frac{1}{{\mathrm{Q}}_{\mathrm{e}}}+{\mathrm{k}}_{2}\mathrm{t}$$

where Q_e_ (mg g^−1^) refers the adsorption capacity at equilibrium; k_1_ (min^−1^) and k_2_ (g mg^−1^ min^−1^) are the adsorption rate constant for pseudo-first order and pseudo-second order respectively; t (min) is the contact time. Table 4 summarizes the kinetics parameters and regression coefficients obtained from data curation of the experimental results. The calculated values from pseudo-second order model are in lines with those from practical experiments, implying that the adsorption of phenol over as-prepared carbon materials is fitted by a pseudo-second order model. Pseudo second order model rate constant (k_2_) determined by PSO model exhibited the following order in terms of the type of the activator: KOH > H_2_O > K_2_CO_3_. This trend indicated that the carbon materials that activated with KOH have higher adsorption rate for phenol removal.

**Table 4:** Various kinetics parameters for the adsorption of phenol over as-prepared porous carbon materials under the optimized conditions of phenol dose of 100 mg L^−1^, temperature of 25 °C, and pH of 4.

Sample	Pseudo-first order			Pseudo-second order		
	Q_e_ (mg g^−1^)	k_1_ (min^−1^)	R^2^	Q_e_ (mg g^−1^)	k_2_ (g mg^−1^ min^−1^)	R^2^
BU_AC_H_2_O_900	137.03	0.151	0.99	142.86	0.003	0.99
BU_AC_k_2_CO_3__700	109.48	0.111	0.99	116.28	0.002	0.99
BU_AC_KOH_700	102.12	0.183	0.99	104.17	0.005	0.99
BS_AC_H_2_O_900	122.64	0.160	0.98	125.00	0.006	0.99
BS_AC_k_2_CO_3__700	83.29	0.171	0.99	82.64	0.013	0.99
BS_AC_KOH_700	89.08	0.370	0.93	89.24	0.020	0.99
SD_AC_H_2_O_900	158.96	0.191	0.99	163.93	0.004	0.99
SD_AC_k_2_CO_3__700	123.55	0.191	0.99	131.58	0.003	0.99
SD_AC_KOH_700	92.13	0.194	0.99	99.00	0.005	0.99

#### Adsorption isotherm

The possible adsorption isothermal model of obtained data was determined by fitting by Langmuir and Freundlich for 50–250 mg L^−1^ solution of phenol at 25 °C. The expressions describe Langmuir (Eq. 13) and Freundlich (Eq. 13) models are given by the following equation:

13 $$\frac{{\mathrm{C}}_{\mathrm{e}}}{{\mathrm{Q}}_{\mathrm{e}}}=\frac{{\mathrm{C}}_{\mathrm{e}}}{{\mathrm{Q}}_{\mathrm{max}}}+\frac{1}{{\mathrm{K}}_{\mathrm{L}}{\mathrm{Q}}_{\mathrm{max}}}$$

14 $${\mathrm{logQ}}_{\mathrm{e}}={\mathrm{logK}}_{\mathrm{F}}+\frac{1}{\mathrm{n}}{\mathrm{logC}}_{\mathrm{e}}$$

where Q_max_, K_L_, n, and K_F_ are maximum adsorption capacity, Langmuir constant and Freundlich constants. Table 5 summarizes the Langmuir and Freundlich parameters and regression coefficients obtained from data curation of the experimental results. As shown, the adsorption behavior of the as-prepared carbon sample for phenol is well match with the Langmuir model (R^2^ = 0.99), indicating that phenol is effectively adsorbed onto AC as a monolayer. The Langmuir isothermal hypothesis can be summarized as follows: (1) The adsorption process cannot occur outside the first layer, (2) all available sites on the surface of the adsorbent are equivalent and absorb one molecule of the adsorbent^52^. In addition, the calculated K_L_ values was in the range of 0.001–0.036, implied a favorable adsorption of phenol molecules upon the optimized parameters. According to the obtained K_L_ values, the samples showed the following trend: H_2_O > K_2_CO_3_ > KOH.

**Table 5:** Langmuir and Freundlich parameters for the adsorption of phenol over as-prepared porous carbon materials under the optimized conditions of phenol dose of 100 mg L^−1^, temperature of 25 °C, and pH of 4.

Sample	Langmuir model			Freundlich model		
	Q_max_	K_L_	R^2^	K_F_	1/n	R^2^
BU_AC_H_2_O_900	248.14	0.029	0.99	0.405	27.68	0.98
BU_AC_k_2_CO_3__700	185.19	0.028	0.98	0.368	23.58	0.99
BU_AC_KOH_700	206.61	0.024	0.96	0.445	18.25	0.91
BS_AC_H_2_O_900	180.50	0.101	0.99	0.198	65.07	0.94
BS_AC_k_2_CO_3__700	184.84	0.034	0.98	0.257	42.09	0.98
BS_AC_KOH_700	149.48	0.001	0.95	0.514	6.32	0.98
SD_AC_H_2_O_900	290.69	0.029	0.99	0.351	42.54	0.99
SD_AC_k_2_CO_3__700	215.52	0.019	0.93	0.406	20.37	0.99
SD_AC_KOH_700	146.20	0.036	0.96	0.226	38.17	0.83

#### Regeneration and mechanism

**Figure 12:**
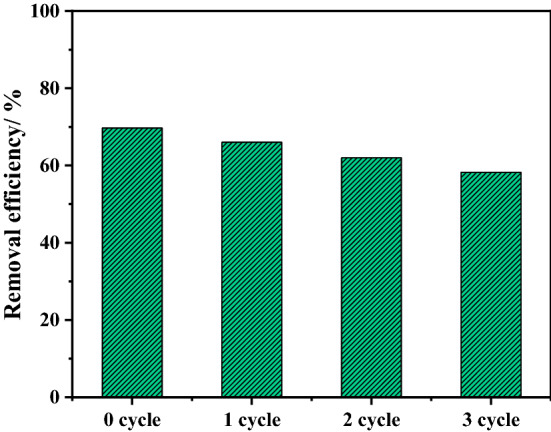
Reusability of SD_AC_H_2_O adsorbent under the adsorption conditions of phenol dose of 100 ppm, temperature of 25 °C, and pH of 4.

In order to regenerate activated carbon sample for further uses, the used AC material was put into 50 mL of the hot water (at 90 °C) and shaken for about 2 h. The regenerated AC was then washed with distilled water and dried at 105 °C^53^. It is clearly observed that the efficiency of the adsorption of phenol molecules gradually decreased after reusing the activated carbon. After three cycles of use Fig. 12, the efficiency of carbon adsorption decreased from 69 to 58%. This can be attributed to the deformation of the absorbent material when used again or due to the saturation of the surface of the adsorbents^54^.

There are many possible interactions between phenol molecules and activated carbon that can derive the adsorption mechanism, namely: electrostatic interaction, π–π interaction, electron donor–acceptor mechanism, and hydrogen bond^55^. As we mentioned above, when the pH of the solution is less than zero-point charge of carbon, the surface charge of carbon is positive, and the phenol molecules maintain un-dissociated under these applied conditions. This leads to electrostatic attraction between the surface of the positively charged active carbon and the electron-rich benzene ring of phenol. It is also possible for a charge transfer to occur from the π-electrons of phenol and the π -electrons of AC, which is called π–π interaction. Due to the presence of functional groups on the surface of the AC, the aromatic phenol ring can act as an electron acceptor, while the functional groups on the surface of carbon acts as an electron donor, this is called electron donor–acceptor mechanism. Finally, –OH group of phenol may form a hydrogen bond with surface of AC at different positions.

## Conclusion

Herein, the fabrication of highly efficient porous carbon materials derived from lignocellulosic wastes have been investigated. Different activators (water vapor, KOH and K_2_CO_3_) have been applied. In addition to the activation temperature and the chemical agent percentage were optimized. Afterwards, the activity of the prepared AC samples toward phenol and MB removal from an aqueous solution were evaluated. The findings of our study could be concluded into the following points.

1. Increasing the activation temperature efficiently increases the S_BET_ values of the AC in the expense of the carbon yield.
2. Increasing the amount of the activating chemical agent drastically decreases the S_BET_ values.
3. Samples physically activated by H_2_O-steam showed significant performance for the removal of both phenol and MB compared with either KOH or K_2_CO_3_ chemical activators.
4. The kinetics and isotherm studies have showed that, the phenol adsorption was better described by pseudo-second-order reaction and Langmuir isotherm, respectively.

In conclusion, the study reveals an easy-to-implement scheme to prepare highly porous activated carbon from lignocellulosic waste materials for efficient application in tertiary treatments units.

## Supplementary Information

Supplementary Information.

## Data Availability

All data generated or analyzed during this study are included in this published article [and its supplementary information files].

## References

[1] Naguib DM, Badawy NM (2020). Phenol removal from wastewater using waste products. J. Environ. Chem. Eng..

[2] Al Bsoul A (2021). Efficient removal of phenol compounds from water environment using Ziziphus leaves adsorbent. Sci. Total Environ..

[3] Peng H, Zou C, Wang C, Tang W, Zhou J (2020). The effective removal of phenol from aqueous solution via adsorption on CS/β-CD/CTA multicomponent adsorbent and its application for COD degradation of drilling wastewater. Environ. Sci. Pollut. Res..

[4] Vaiano V (2018). Enhanced photocatalytic removal of phenol from aqueous solutions using ZnO modified with Ag. Appl. Catal. B Environ..

[5] Mohamad Said KA (2021). Zinc ferrite migration dependence on magnetic induce membrane for phenol removal: Adsorption reaction and diffusion study. J. Environ. Chem. Eng..

[6] Saleh TA, Elsharif AM, Asiri S, Mohammed A-RI, Dafalla H (2020). Synthesis of carbon nanotubes grafted with copolymer of acrylic acid and acrylamide for phenol removal. Environ. Nanotechnol. Monit. Manag..

[7] Sayyed AJ (2021). Cellulose-based nanomaterials for water and wastewater treatments: A review. J. Environ. Chem. Eng..

[8] Hao Z, Wang C, Yan Z, Jiang H, Xu H (2018). Magnetic particles modification of coconut shell-derived activated carbon and biochar for effective removal of phenol from water. Chemosphere.

[9] Hussain SN, Roberts EPL, Asghar HMA, Campen AK, Brown NW (2013). Oxidation of phenol and the adsorption of breakdown products using a graphite adsorbent with electrochemical regeneration. Electrochim. Acta.

[10] Sher F, Malik A, Liu H (2013). Industrial polymer effluent treatment by chemical coagulation and flocculation. J. Environ. Chem. Eng..

[11] Zagklis DP, Vavouraki AI, Kornaros ME, Paraskeva CA (2015). Purification of olive mill wastewater phenols through membrane filtration and resin adsorption/desorption. J. Hazard. Mater..

[12] Karri RR, Jayakumar NS, Sahu JN (2017). Modelling of fluidised-bed reactor by differential evolution optimization for phenol removal using coconut shells based activated carbon. J. Mol. Liq..

[13] Lütke SF (2019). Preparation of activated carbon from black wattle bark waste and its application for phenol adsorption. J. Environ. Chem. Eng..

[14] Ramezanipour Penchah H, Ghaemi A, Jafari F (2021). Piperazine-modified activated carbon as a novel adsorbent for CO_2_ capture: Modeling and characterization. Environ. Sci. Pollut. Res..

[15] Galletti C, Dosa M, Russo N, Fino D (2021). Zn^2+^ and Cd^2+^ removal from wastewater using clinoptilolite as adsorbent. Environ. Sci. Pollut. Res..

[16] Wang Y (2020). Lanthanum hydroxide: A highly efficient and selective adsorbent for arsenate removal from aqueous solution. Environ. Sci. Pollut. Res..

[17] Wang H, Xu J, Liu X, Sheng L (2021). Preparation of straw activated carbon and its application in wastewater treatment: A review. J. Clean. Prod..

[18] Mohamad Nor N, Lau LC, Lee KT, Mohamed AR (2013). Synthesis of activated carbon from lignocellulosic biomass and its applications in air pollution control—A review. J. Environ. Chem. Eng..

[19] González-García P (2018). Activated carbon from lignocellulosics precursors: A review of the synthesis methods, characterization techniques and applications. Renew. Sustain. Energy Rev..

[20] Heidarinejad Z (2020). Methods for preparation and activation of activated carbon: A review. Environ. Chem. Lett..

[21] Jawad AH, Ishak MAM, Farhan AM, Ismail K (2017). Response surface methodology approach for optimization of color removal and COD reduction of methylene blue using microwave-induced NaOH activated carbon from biomass waste. Water Treat.

[22] Jawad AH, Abdulhameed AS (2020). Statistical modeling of methylene blue dye adsorption by high surface area mesoporous activated carbon from bamboo chip using KOH-assisted thermal activation. Energy Ecol. Environ..

[23] Abdulhameed AS (2021). Statistical modeling and mechanistic pathway for methylene blue dye removal by high surface area and mesoporous grass-based activated carbon using K2CO3 activator. J. Environ. Chem. Eng..

[24] Jawad AH (2021). High surface area and mesoporous activated carbon from KOH-activated dragon fruit peels for methylene blue dye adsorption: Optimization and mechanism study. Chin. J. Chem. Eng..

[25] Jawad AH, Mehdi ZS, Ishak MAM, Ismail K (2018). Large surface area activated carbon from low-rank coal via microwave-assisted KOH activation for methylene blue adsorption. Desalin Water Treat.

[26] Tabassum M, Bardhan M, Novera TM, Islam M, HadiJ awad A (2020). NaOH-activated betel nut husk hydrochar for efficient adsorption of methylene blue dye. Water Air Soil Pollut..

[27] Jawad AH (2021). Microporous activated carbon developed from KOH activated biomass waste: surface mechanistic study of methylene blue dye adsorption. Water Sci. Technol..

[28] Sluiter A (2008). Determination of structural carbohydrates and lignin in biomass. Lab. Anal. Proced..

[29] Danish M, Ahmad T (2018). A review on utilization of wood biomass as a sustainable precursor for activated carbon production and application. Renew. Sustain. Energy Rev..

[30] Xue Y, Du C, Wu Z, Zhang L (2018). Relationship of cellulose and lignin contents in biomass to the structure and RB-19 adsorption behavior of activated carbon. New J. Chem..

[31] Zhou J, Luo A, Zhao Y (2018). Preparation and characterisation of activated carbon from waste tea by physical activation using steam. J. Air Waste Manage. Assoc..

[32] González-García P, Gamboa-González S, Andrade Martínez I, Hernández-Quiroz T (2020). Preparation of activated carbon from water hyacinth stems by chemical activation with K2CO3 and its performance as adsorbent of sodium naproxen. Environ. Prog. Sustain. Energy.

[33] da Paixão Cansado IP, Belo CR, Mira Mourão PA (2019). Pesticides abatement using activated carbon produced from a mixture of synthetic polymers by chemical activation with KOH and K2CO3. Environ. Nanotechnol. Monit. Manag..

[34] El-Bery HM, Salah MR, Ahmed SM, Soliman SA (2021). Efficient non-metal based conducting polymers for photocatalytic hydrogen production: Comparative study between polyaniline, polypyrrole and PEDOT. RSC Adv..

[35] Mahmoud AH (2021). Latex-bearing plant (*Calotropis procera*) as a biorefinery for bioethanol production. Biomass Convers. Biorefinery..

[36] El-Bery HM, Abdelhamid HN (2021). Photocatalytic hydrogen generation via water splitting using ZIF-67 derived Co3O4@ C/TiO2. J. Environ. Chem. Eng..

[37] Saleh MR, Ahmed SM, Soliman SA, El-Bery HM (2022). Facile construction of self-assembled Cu@polyaniline nanocomposite as an efficient noble-metal free cocatalyst for boosting photocatalytic hydrogen production. Int. J. Hydrogen Energy.

[38] Saleh MR, El-Bery HM (2022). Unraveling novel Cu/CuxP@N-doped C composite as effective cocatalyst for photocatalytic hydrogen production under UV and visible irradiation. Appl. Surf. Sci..

[39] Hou J (2020). Sorghum-waste-derived high-surface area KOH-activated porous carbon for highly efficient methylene blue and Pb(II) removal. ACS Omega.

[40] Ali AM (2021). Synthesis, characterization and photoelectric properties of Fe2O3 incorporated TiO2 photocatalyst nanocomposites. Catalysts.

[41] Mishra S, Yadav SS, Rawat S, Singh J, Koduru JR (2019). Corn husk derived magnetized activated carbon for the removal of phenol and para-nitrophenol from aqueous solution: Interaction mechanism, insights on adsorbent characteristics, and isothermal, kinetic and thermodynamic properties. J. Environ. Manage..

[42] Nirmala G, Murugesan T, Rambabu K, Sathiyanarayanan K, Show PL (2021). Adsorptive removal of phenol using banyan root activated carbon. Chem. Eng. Commun..

[43] Rambabu K (2019). Activated carbon from date seeds for chromium removal in aqueous solution. Desalin. Water Treat..

[44] Makrigianni V, Giannakas A, Deligiannakis Y, Konstantinou I (2015). Adsorption of phenol and methylene blue from aqueous solutions by pyrolytic tire char: Equilibrium and kinetic studies. J. Environ. Chem. Eng..

[45] Sierra I, Iriarte-Velasco U, Cepeda EA, Gamero M, Aguayo AT (2016). Preparation of carbon-based adsorbents from the pyrolysis of sewage sludge with CO_2_. Investigation of the acid washing procedure. Desalin. Water Treat..

[46] Gokce Y, Aktas Z (2014). Nitric acid modification of activated carbon produced from waste tea and adsorption of methylene blue and phenol. Appl. Surf. Sci..

[47] Kumar A, Jena HM (2016). Removal of methylene blue and phenol onto prepared activated carbon from Fox nutshell by chemical activation in batch and fixed-bed column. J. Clean. Prod..

[48] da Gama BMV (2018). Mono and binary component adsorption of phenol and cadmium using adsorbent derived from peanut shells. J. Clean. Prod..

[49] Zhang D, Huo P, Liu W (2016). Behavior of phenol adsorption on thermal modified activated carbon. Chin. J. Chem. Eng..

[50] Rodrigues LA, da Silva MLCP, Alvarez-Mendes MO, do Reis Coutinho A, Thim GP (2011). Phenol removal from aqueous solution by activated carbon produced from avocado kernel seeds. Chem. Eng. J..

[51] Singh KP, Malik A, Sinha S, Ojha P (2008). Liquid-phase adsorption of phenols using activated carbons derived from agricultural waste material. J. Hazard. Mater..

[52] Egbosiuba TC (2020). Ultrasonic enhanced adsorption of methylene blue onto the optimized surface area of activated carbon: Adsorption isotherm, kinetics and thermodynamics. Chem. Eng. Res. Des..

[53] El-Naas MH, Al-Zuhair S, Alhaija MA (2010). Removal of phenol from petroleum refinery wastewater through adsorption on date-pit activated carbon. Chem. Eng. J..

[54] Baytar O, Şahin Ö, Horoz S, Kutluay S (2020). High-performance gas-phase adsorption of benzene and toluene on activated carbon: Response surface optimization, reusability, equilibrium, kinetic, and competitive adsorption studies. Environ. Sci. Pollut. Res..

[55] Supong A (2020). Experimental and theoretical insight into the adsorption of phenol and 2,4-dinitrophenol onto Tithonia diversifolia activated carbon. Appl. Surf. Sci..

