# Supplementary material for: High adsorption capacity of phenol and methylene blue using activated carbon derived from lignocellulosic agriculture wastes
Source: Sci Rep. 2022 Mar 31;12:5499. doi: 10.1038/s41598-022-09475-4 (PMC8971380; doi:10.1038/s41598-022-09475-4)
Supplement: Supplementary file 1 — Supplementary Information. [file 41598_2022_9475_MOESM1_ESM.docx]

**High adsorption capacity of phenol and methylene blue using activated carbon derived from lignocellulosic agriculture wastes**

**Haitham M. El-Bery*, Moushira Saleh, Reem A. El-Gendy, Mahmoud R. Saleh*, Safinaz M. Thabet**

Advanced Multifunctional Materials Laboratory, Chemistry Department, Faculty of Science, Assiut University, Assiut 71515, Egypt.

***Corresponding Author Email:** [**Haitham.El-Bery@aun.edu.eg**](mailto:Haitham.El-Bery@aun.edu.eg)**;** [**mahmoudrabie@aun.edu.eg**](mailto:mahmoudrabie@aun.edu.eg)

**Fig. S1.** Photographic images of bagasse un-sieved (BU), bagasse sieved (BS) and sawdust (SD).

**Fig. S2.** Schematic diagram of the experimental setup: (a) carbonization test, (b) water activation test.

**Fig. S3**. UV-vis spectrum of phenol solution at different concentrations (a). The calibration curve calibration curve constructed based on Beer’s-Lambert law (b).

**Fig. S4**. UV-vis spectrum of methylene blue solution at different concentrations (a). The calibration curve calibration curve constructed based on Beer’s-Lambert law (b).

**Fig. S5.** TGA curves of BS, BU, and SD measured from room temperature until 1000^o^C with heating rate of 10^o^C/min.

**Fig. S6**. UV-vis spectrum of methylene blue solution at different time intervals for optimized samples.

**Fig. S7**. UV-vis spectrum of phenol solution at different time intervals for optimized samples.

**Table S1:** Raw lignocellulosic materials analysis

| Analysis/  Samples | Cellulose  (%) | Hemicellulose  (%) | Lignin  (%) | Moisture  (M) (%) | Volatile matters  (VC) (%) | Ash  (AC) (%) | Fixed carbon  (FC) (%) |
| --- | --- | --- | --- | --- | --- | --- | --- |
| BU | 45.6 | 17.0 | 24.4 | 6 | 87.1 | 6.5 | 6.4 |
| BS | 39.2 | 24.0 | 17.9 | 6.1 | 88.3 | 4.3 | 7.4 |
| SD | 35.6 | 15.2 | 42.4 | 7 | 86.8 | 4.7 | 8.56 |

**Table S2:** Surface texture properties BU-car sample activated with various activators, water vapor, K_2_CO_3_, and KOH, at different temperatures and different weight of chemical activators

| Sample | BET method | | t method | | BJH method | |
| --- | --- | --- | --- | --- | --- | --- |
|  | Surface area (m^2^/g) | Micropore volume (cm^3^/g) | Micropore area (m^2^/g) | External area (m^2^/g) | Pore volume (cm^3^/g) | Pore radius (A^o^) |
| BU_car | 664 | 0.34 | 646 | 18 | 0.03 | 18.0 |
| BU_AC_H_2_O_800 | 987 | 0.46 | 928 | 59 | 0.11 | 18.1 |
| BU_AC_H_2_O_850 | 1346 | 0.63 | 1180 | 167 | 0.27 | 18.1 |
| BU_AC_H_2_O_900 | 1470 | 0.69 | 1332 | 138 | 0.25 | 18.2 |
| BU_AC_H_2_O_950 | 1771 | 0.72 | 1399 | 372 | 0.68 | 18.0 |
| BU_AC_K_2_CO_3__700 | 1392 | 0.68 | 1332 | 61 | 0.09 | 18.0 |
| BU_AC_K_2_CO_3__750 | 1935 | 0.92 | 1831 | 104 | 0.10 | 18.2 |
| BU_AC_K_2_CO_3__800 | 2120 | 1.01 | 2007 | 113 | 0.16 | 15.7 |
| BU_AC_KOH_700 | 1463 | 0.70 | 1392 | 71 | 0.11 | 18.1 |
| BU_AC_KOH_750 | 2088 | 0.96 | 1938 | 149 | 0.19 | 15.8 |
| BU_AC_KOH_800 | 2490 | 1.13 | 2262 | 229 | 0.34 | 15.7 |
| BU_AC_K_2_CO_3_ (1:1) | 1392 | 0.68 | 1332 | 61 | 0.09 | 18.0 |
| BU_AC_K_2_CO_3_ (1:2) | 897 | 0.44 | 856 | 41 | 0.06 | 18.1 |
| BU_AC_K_2_CO_3_ (1:3) | 529 | 0.354 | 505 | 24 | 0.04 | 18.1 |
| BU_AC_KOH (1:1) | 1463 | 0.70 | 1392 | 71 | 0.11 | 18.1 |
| BU_AC_KOH (1:2) | 990 | 0.46 | 924 | 65 | 0.08 | 15.7 |
| BU_AC_KOH (1:3) | 826 | 0.38 | 772 | 54 | 0.07 | 15.7 |

**Table S3:** Comparison of adsorption efficiency of phenol, specific surface area and yield of different carbon samples activated by chemical and physical processes

| Sample | Yield  (%) | Specific surface area  (m^2^ g) | Adsorption capacity  (mg g^-1^) |
| --- | --- | --- | --- |
| BU_car | -- | 664 | 36.4 |
| BU_AC_H_2_O_900 | 42 | 1470 | 142.2 |
| BU_AC_k_2_CO_3__700 | 62 | 1392 | 119.8 |
| BU_AC_KOH_700 | 61 | 1463 | 105.6 |
| BS_car | -- | 243 | 37.8 |
| BS_AC_H_2_O_900 | 53 | 1228 | 127.7 |
| BS_AC_k_2_CO_3__700 | 41 | 1482 | 85.2 |
| BS_AC_KOH_700 | 31 | 1204 | 88.0 |
| SD_car | -- | 534 | 29.4 |
| SD_AC_H_2_O_900 | 53 | 1053 | 158.8 |
| SD_AC_k_2_CO_3__700 | 67 | 1528 | 123.4 |
| SD_AC_KOH_700 | 50 | 1544 | 96.8 |
